# Cytochromes P450 and P-Glycoprotein Phenotypic Assessment to Optimize Psychotropic Pharmacotherapy: A Retrospective Analysis of Four Years of Practice in Psychiatry

**DOI:** 10.3390/jpm12111869

**Published:** 2022-11-08

**Authors:** Clément Delage, Léa Darnaud, Bruno Etain, Marina Vignes, Tu-Ky Ly, Alexia Frapsauce, Marc Veyrier, Marine Delavest, Emeline Marlinge, Vincent Hennion, Manon Meyrel, Aude Jacob, Margot Chouchana, Julie Smati, Guillaume Pataud, Nihel Khoudour, Jean-Eudes Fontan, Laurence Labat, Frank Bellivier, Célia Lloret-Linares, Xavier Declèves, Vanessa Bloch

**Affiliations:** 1 Service Pharmacie, AP-HP, Hôpital Lariboisière Fernand Widal, F-75010 Paris, France; 2 Université Paris Cité, Inserm UMRS-1144, Optimisation Thérapeutique en Neuropsychopharmacologie, F-75006 Paris, France; 3 Université Paris Cité, F-75006 Paris, France; 4 Service Laboratoire de Biologie du Médicament et Toxicologie, AP-HP, Hôpital Cochin, F-75014 Paris, France; 5 Département de Psychiatrie et Médecine Addictologique, AP-HP, Hôpital Lariboisière Fernand Widal, F-75010 Paris, France; 6 Service Laboratoire de Toxicologie, AP-HP, Hôpital Lariboisière Fernand Widal, F-75010 Paris, France; 7 Ramsay Santé-Hôpital Privé Pays de Savoie, F-74100 Annemasse, France

**Keywords:** phenotyping, cytochromes P450, personalized medicine, therapeutic optimization, psychiatry, P-glycoprotein

## Abstract

Altered cytochromes P450 enzymes (CYP) and P-glycoprotein transporter (P-gp) activity may explain variabilities in drug response. In this study, we analyzed four years of phenotypic assessments of CYP/P-gp activities to optimize pharmacotherapy in psychiatry. A low-dose probe cocktail was administered to evaluate CYP1A2, 2B6, 2D6, 2C9, 2C19, 3A4, and P-gp activities using the probe/metabolite concentration ratio in blood or the AUC. A therapeutic adjustment was suggested depending on the phenotyping results. From January 2017 to June 2021, we performed 32 phenotypings, 10 for adverse drug reaction, 6 for non-response, and 16 for both reasons. Depending on the CYP/P-gp evaluated, only 23% to 56% of patients had normal activity. Activity was decreased in up to 57% and increased in up to 60% of cases, depending on the CYP/P-gp evaluated. In 11/32 cases (34%), the therapeutic problem was attributable to the patient’s metabolic profile. In 10/32 cases (31%), phenotyping excluded the metabolic profile as the cause of the therapeutic problem. For all ten individuals for which we had follow-up information, phenotyping allowed us to clearly state or clearly exclude the metabolic profile as a possible cause of therapeutic failure. Among them, seven showed a clinical improvement after dosage adaptation, or drug or pharmacological class switching. Our study confirmed the interest of CYP and P-gp phenotyping for therapeutic optimization in psychiatry.

## 1. Introduction

Despite the numerous drugs available to treat mood disorders, therapeutic failures are common in psychiatry. It is estimated that only 25 to 50% of individuals respond to the first line of pharmacotherapy [1,2]. There are many possible causes of treatment-resistant depression, such as inadequate pharmacological targets, inadequate treatment dosage and/or duration, neuroprotective agent intake, inadequate diagnosis, lack of compliance, etc. [3] Among them, interindividual variability in drug plasma concentration is a possible reason for drug failure. At the same oral dose, plasma concentration can vary by a factor of 20 due to different pharmacokinetic parameters: primarily drug metabolism and elimination [4]. Thus, therapeutic drug monitoring (TDM) is recommended for most psychotropic drugs due to their narrow therapeutic margins [4]. However, when facing abnormal plasma levels or recurrent drug failures, such as non-response or adverse drug reactions, TDM has limits in identifying the origin of the therapeutic failure.

Cytochromes P450 (CYP) are the main drug-metabolizing enzymes [5]. They include many different isoforms, but only a few account for most drug metabolisms, such as CYP1A2, CYP2B6, CYP2C9, CYP2C19, CYP2D6, and CYP3A4, which are responsible for the metabolization of 90% of all drugs [6]. In addition to CYP, P-glycoprotein (P-gp) is an efflux membrane transporter located in enterocytes of the small intestine, the biliary pole of hepatocytes, the apical membrane of renal tubule epithelial cells, and in brain microvessel endothelial cells [7]. P-gp is responsible for the extrusion of molecules from the central nervous system to the blood and from the blood to the gastrointestinal lumen, bile, and urine. Thus, an increase in CYP or P-gp activity decreases the blood concentration of their substrates, which may lead to a lack of therapeutic effect. Conversely, a decrease in a CYP or P-gp activity increases the blood concentration of their substrates, which may lead to an accumulation of the drug and the potential for adverse drug reactions. This phenomenon is reversed in the case of a prodrug, which is active after metabolization. It is well known that CYP and P-gp genotypes vary substantially both between ethnic groups and within the same group [8]. Thus, these genetic polymorphisms represent a major factor in interindividual variability in drug response.

Evaluating CYP and P-gp activity by genotyping their encoding genes has proved its efficacy in predicting drug metabolization [9] and increasing the effectiveness of antidepressant therapies [10]. However, genotype does not always correlate exactly with plasma concentration [11,12], and phenoconversions (when the measured phenotype is different from the genotype) occur in between 30 and 60% of cases [10,13,14,15,16,17]. Phenoconversions may be due to different factors, among which are physiological situations [18] and environmental factors such as smoking, diet and drug intake [8]. Then, the phenotypic assessment of CYP and P-gp activity appears a more effective tool than genotyping for evaluating the metabolic profile of individuals [19]. One method of CYP and P-gp phenotypic assessment consists of a low-dose probe drug “cocktail” administration, together with the quantitative blood analysis of probes and their metabolites [20,21,22]. Some authors have described a potential clinical application of phenotyping for therapeutic optimization to anti-HIV and anti-tuberculosis drugs [23], anticoagulants [24], analgesics [17], anticancer agents [25], and psychotropic drugs [26], but the therapeutic and clinical impact of phenotyping remains largely unexplored, especially in psychiatry.

In our university-based hospital, we implemented a CYP/P-gp phenotypic assessment in the psychiatric department as a tool to optimize pharmacotherapy when physicians faced therapeutic problems, such as adverse drug reactions or non-response to psychotropic drugs. In this article, we retrospectively describe the data and outcomes of four years of phenotyping in individuals with mood disorders.

## 2. Materials and Methods

### 2.1. Patients and Phenotyping Decision

Phenotyping took place in Fernand Widal Hospital (Assistance Publique Hôpitaux de Paris (APHP), Paris, France). Physicians at in- and out-patient units facing adverse drug reactions or drug non-response for a given individual contacted the pharmacy unit to investigate the implication of the metabolic profile for this therapeutic problem. Based on a comprehensive medical and therapeutic history (past and current treatments) and previous drug-level blood tests, the pharmacist reported possible drug–drug interactions and CYP/P-gp alternative profiles that might explain this problem. This report was accompanied by recommendations with regard to therapeutic drug monitoring and/or CYP/P-gp phenotyping. A phenotyping decision was made after a multidisciplinary discussion between physicians and pharmacists, and the CYP/P-gp phenotyping to be explored was determined based on the pharmacist’s report. Individuals with documented intolerance or contraindications to one of the probe drugs were excluded from phenotyping.

### 2.2. Ethics

Ethical approval was obtained from the French Ethics Committee “Comité de Protection des Personnes Sud Méditerranée” and numbered as ID-RCB-2017-A00685-48. All patients signed a consent form before starting the phenotyping protocol.

### 2.3. Cytochrome and P-Glycoprotein Phenotyping

Phenotyping procedures took place in a psychiatric hospitalization unit or day hospital. CYP/P-gp activities were assessed using a cocktail approach previously described by Bosilkovska et al. [27,28] and called the “Geneva Cocktail”. The cocktail approach consists of administering several probe drugs, each one being metabolized by a specific CYP or substrate of the P-gp. Measuring the plasma level of both probe drugs and metabolites at a specific time allows us to determine the activity of the CYP. As a transporter, P-gp activity is assessed with probe drug plasma level only. The “Geneva Cocktail” has the advantage of containing substrates completely specific for each CYP of interest and the P-gp, and with low doses does not produce side effects. It is composed of caffeine 50 mg for CYP1A2, bupropion 150 mg for CYP2B6, omeprazole 10 mg for CYP2C19, flurbiprofen 50 mg for CYP2C9, dextromethorphan 10 mg for CYP2D6, midazolam 1 mg for CYP3A4 and fexofenadine 120 mg for P-gp. Full details of the cocktail (probe drugs, metabolites, CYP/P-gp-targeted, galenic form, dosage, and route of administration) can be found in Supplemental Data Table S1. The cocktail was adapted depending on the CYP/P-gp explored.

**Figure 1:**
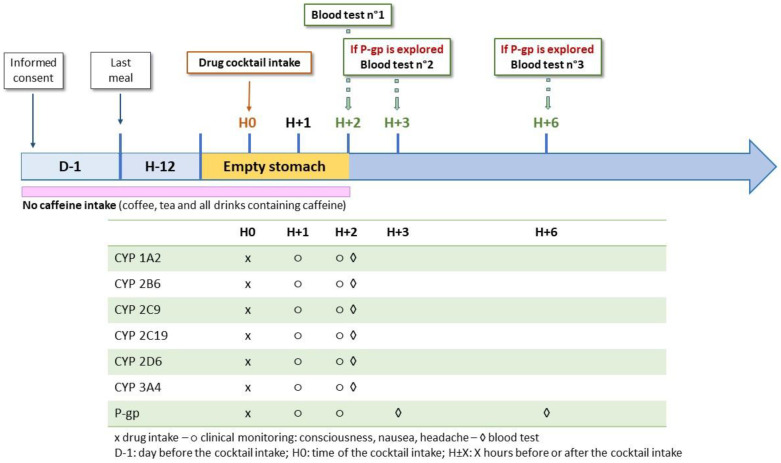
Phenotyping protocol.

Individuals had to fast overnight before cocktail intake. They had to keep fasting until the first blood test and to delay their eventual morning drug intake until immediately afterwards. As CYP1A2 activity was assessed with caffeine, patients were asked to avoid caffeine intake (coffee, cola beverages, tea, etc.) for 24 h before the cocktail intake when this CYP was assessed to avoid biasing plasma measurements (Figure 1). The cocktail administration was supervised by a nurse. Blood samples were collected into a lithium heparin tube by a nurse and stored at room temperature (<25 °C) before shipment to the laboratory (<24 h). If not processed immediately, samples were stored between −15 °C and −25 °C awaiting analysis (<3 weeks).

Administered drugs and their metabolites were quantified in plasma by the toxicology laboratory in Cochin Hospital (AP-HP, Paris, France). The quantification was performed using a previously described HPLC-MS/MS method [27,29]. CYP activities were assessed by specific metabolite/probe concentration ratios (metabolic ratios (MRs)) determined 2 h after cocktail administration based on previous studies [27]. P-gp activity was assessed through the estimation of the area under the curve of the probe concentration using dosing at 3 and 6 h after the cocktail administration (AUC_0–6h_), as previously described [27,29]. P-gp and CYP activities, as measured by AUC_0–6h_ and MRs, respectively, were classified as decreased, normal, or increased. CYP2D6 activity was classified as ultrarapid metabolizer (UM), extensive metabolizer (EM), intermediate metabolizer (IM), or poor metabolizer (PM). These classifications were based on ranges previously determined in a healthy population by Bosilkovksa et al. [27,29]. To ease the readability of the results, CYP2D6 UM and EM were in some cases considered as an increased phenotype, IM as a normal phenotype, and PM as a decreased phenotype.

### 2.4. Clinical Application

Based on the CYP/P-gp phenotype and considering current and past treatment, the pharmacist provided a therapeutic opinion on the involvement of the metabolic profile in the therapeutic problem. The report was accompanied by therapeutic advice such as drug switching or dosage adaptation. The report was delivered by the pharmacist to the physician during a dedicated appointment to discuss the different therapeutic options for the patients.

### 2.5. Retrospectively Analyzed Data

To assess our phenotyping activity, we retrospectively analyzed the following data:

- Patient characteristics: age, gender, alcohol, tobacco and grapefruit consumption, and kidney and hepatic function.
- Clinical context and therapeutic problem: adverse drug reaction with one or multiple drugs at usual doses (ADR), no therapeutic response to one or multiple drugs (NR), or adverse drug reaction for some drugs and non-response for others (ADR+NR).
- Involved drugs: past and current drugs for which the physician questioned the implication of the metabolic profile in the therapeutic problem encountered, and the CYP/P-gp of which they were major or minor substrates [30].
- Current treatment: drugs prescribed to the patient at the moment of phenotyping, their dosage, their possible inhibiting or inducing properties on CYP/P-gp, and their potency of action (weak or potent) [30].
- CYP/P-gp activity: increased/UM, EM, normal/IM or decreased/PM.
- Answer to the therapeutic problem: the metabolic profile was (Y) or was not (N) the cause of the therapeutic problem; could explain partially the therapeutic problem for some drugs only (P); or was not interpretable (NI) due to CYP/P-gp inducers or inhibitors or because too many metabolic pathways were involved in the metabolism of the drugs implicated.
- Follow-up: therapeutic adaptations made by the physician and their clinical consequences.

## 3. Results

### 3.1. Population

Between January 2017 and June 2021, 37 phenotyping demands were received. Multidisciplinary discussions resulted in judging phenotyping unnecessary in five cases: four because of the involvement of drugs not metabolized by the CYP/P-gp, and one because of the recent and temporary introduction of a potent inducer. Thus, 32 phenotypings were performed on 31 different patients: 9 men and 22 women (one patient had two phenotypic assessments) (Table 1). Patients were aged from 25 to 75 years, with a mean age of 47.1 ± 14.5 years and median age of 49 years [35–58.5].

Nine patients were smokers and four drank an average of two or more glasses of alcohol per day. None consumed grapefruit or *Hypericum*.

Thirteen phenotypings (41%) were performed during hospitalization, others (n = 19; 59%) were performed in a day hospital. Phenotypings were performed in the context of depressive disorder in 15 cases (47%), bipolar disorder in 12 cases (38%), anxiety–depressive syndrome in 3 cases (9%), schizoaffective disorder in 1 case (3%), and neuropathic pain in 1 case (3%). The first of the two phenotypings performed for the same patients were in the context of depressive disorders. The second was in the context of neuropathic pain. Among the causes of phenotyping, 10 (31%) were due to ADR, 6 (19%) were due to NR, and 16 (50%) were due to ADR+NR (ADR for some drugs and NR for others) (Table 1).

Full details of individual patient characteristics can be found in the supplemental data (Table S2).

### 3.2. Drugs Involved

For each phenotypic assessment, the implication of the metabolic profile was questioned for an average of 7.0 ± 4.8 different drugs taken in the past or currently prescribed. Antidepressants were involved in 25 phenotypings (20 different drugs), antipsychotics in 24 (14 different drugs), anxiolytics in 12 phenotypings (7 different drugs) and antiepileptics in 12 phenotypings (9 different drugs). The molecules most involved in the phenotypings are listed in Table 2 (the full list can be found in the supplemental data—Table S3). These drugs correspond to the drugs the most prescribed in our hospital, as they are the most recommended in international guidelines.

**Figure 2:**
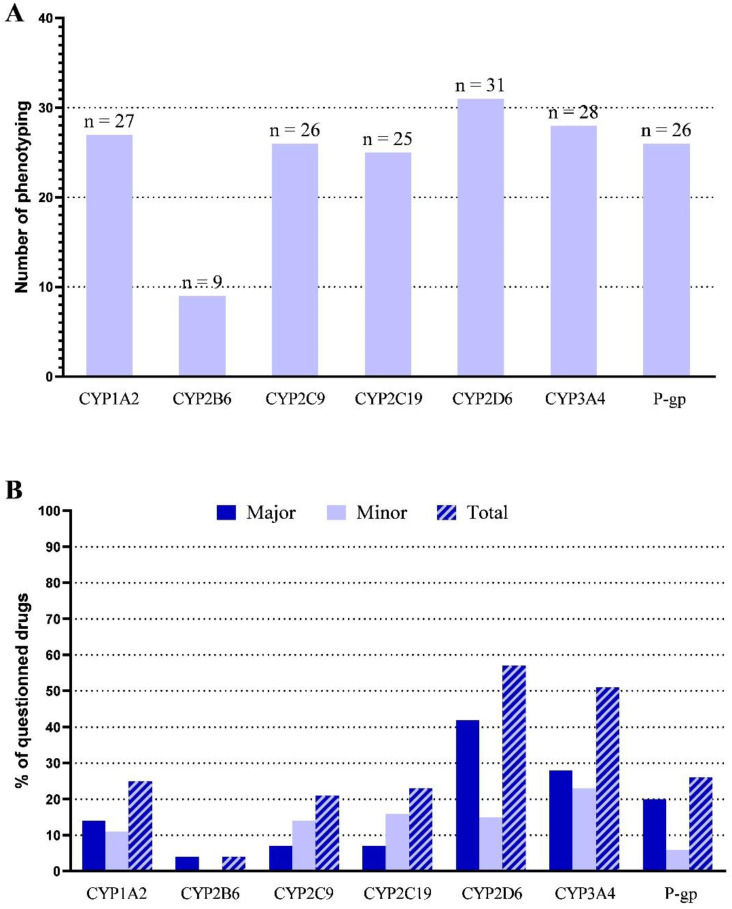
(**A**) Implications of the different CYP/P-gp profiles with the 32 phenotypings and (**B**) proportion of drugs that are minor (light blue) or major (dark blue) substrates of the CYP/P-gp profiles among the 224 cumulated drugs implicated in the therapeutic problem that led to the 32 phenotypic assessments (n = 224 cumulated drugs = sum of all drugs involved in each of the 32 phenotypings; a drug involved in more than one phenotyping was counted cumulatively for as many times as the phenotypings).

CYP2D6 was implicated in almost all phenotypings, unlike 2B6, which was involved in only a few cases. CYP2D6 and CYP3A4 were implicated in the metabolization of the majority of the drugs questioned through phenotyping with 56.7% and 51.3% of the 224 cumulated drugs, respectively (cumulated drugs = sum of all drugs involved in each of the 32 phenotypings; a drug involved in more than one phenotyping was counted cumulatively for as many times as the phenotypings), that were substrates of at least these CYPs (Figure 2 and Supplementary Table S3).

### 3.3. Response to the Therapeutic Problems

Phenotyping indicated a metabolic profile compatible with under- or overdosing that might have caused the therapeutic problem encountered (Y) in 11/32 cases (34%). In 10/32 cases (31%), the phenotyping precluded the metabolic profile as a possible cause of the therapeutic problem (N). Thus, phenotyping gave a clear answer to the physician’s question (Y + N) in 21/32 cases (66%). Conversely, in 7/32 cases (22%), the metabolic profile might have been partially (P) responsible for the therapeutic problem, for some drugs only. The phenotyping was not interpretable (NI) in 4/32 cases (13%) because of the presence of CYP/P-gp inducers or inhibitors, or because too many metabolic pathways were involved in the elimination of the drugs implicated (Table 3).

When phenotyping allowed the involvement of the metabolic profile in the therapeutic problem to be identified (Y), an average of 4.5 ± 2.9 drugs were implicated in the therapeutic problem. Conversely, when the phenotyping did not bring a clear answer to the problem encountered (P + NI), an average of 8.0 ± 5.8 drugs were implicated (Table 3).

### 3.4. Individual CYP/P-gp Activities

Full details of individual CYP activities can be found in the Supplementary Data (Table S2). Among the 32 phenotypings, CYP/P-gp activities were normal in 23% to 56% of the cases, depending on the CYP/P-gp. CYP-P-gp activities were decreased in 0% to 57% of cases and increased in 9% to 60% of cases. (Table 4).

In the presence of an inhibitor (weak or potent), the activity of the corresponding CYP/P-gp was decreased in 13% to 89% of cases. This phenomenon seemed less important with weak inhibitors (13% to 83%) than with potent inhibitors (42% to 100% of cases) (Table 4). A detailed list of all the different CYP/P-gp inhibitors involved and the measured activity of the corresponding CYP/P-gp can be found in Supplementary Table S4. In the presence of a CYP/P-gp inducer (weak or potent), the activity of the CYP/P-gp involved was increased in 6/13 cases (46%), and in only 1/7 cases (14%) in the case of a weak inducer. The detailed list of all the CYP/P-gp inducers and the measured activity of the corresponding CYP/P-gp can be found in Supplementary Table S5.

In 6/7 patients (86%) who were smoking and whose CYP1A2 activity was measured, the activity was increased (none had inhibitor or inducer). In the four individuals that were consuming two or more glasses of alcohol per day, CYP1A2 and CYP3A4 activities were assessed in three and four cases, respectively. CYP1A2 was increased in one patient only, and the two others had normal activity (but one of the patients with normal activity had an inhibitor at the moment of phenotyping). CYP3A4 activity was decreased in 1/4 cases and was normal in 3/4 cases (but two of them had an inhibitor at the moment of phenotyping).

### 3.5. Follow-Up

**Figure 3:**
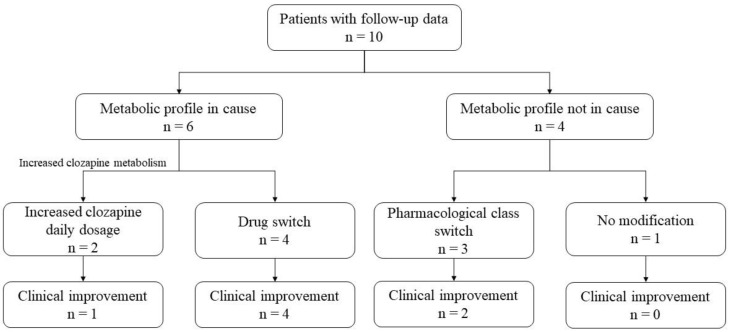
Therapeutic decision and clinical consequences following phenotyping.

Of the 31 patients who had undergone phenotyping, 12 were followed up by a physician at Fernand Widal Hospital after the phenotyping; among them, we obtained follow-up data for ten patients (Figure 3).

The metabolic profile was compatible with an underdosing of clozapine revealed by a plasma test in 2/10 patients. Daily dosages were increased, resulting in clinical improvement in 1/2 patients. When the decreased metabolic status was compatible with the side effects (4/10 patients), a drug switch was performed, resulting in clinical improvement in all patients (4/4) (Figure 3). When the metabolic status was not implicated in the therapeutic problem (4/10 patients), a switch of pharmacological drug class was performed in 3/4 patients, two of whom showed clinical improvement (Figure 3).

## 4. Discussions

The use of CYP/P-gp activity assessment can address two situations: as a complementary tool to TDM to select the medication and adjust the dosage through genotype-based recommendations [31,32], or to investigate a drug failure or an abnormal TDM [16,17]. Genotyping is increasingly being suggested for the former situation through genotype-based recommendations [31,33]. Pharmacogenetics has expanded since its introduction in some countries, and testing panels are marketed to physicians or directly to customers [33]. However, as phenoconversion happens in 30 to 60% of cases [13,14], genotyping is still imprecise unless several allelic variants are analyzed, which is not the case in the marketed tests [34]. Thus, phenotypic assessment appears more effective for investigating therapeutic drug failure [19], but studies assessing its benefits, especially in terms of clinical outcomes, are scarce.

In our hospital, we have been using phenotyping for the past four years to investigate drug failures. In cases of non-response or adverse drug reaction, we proceeded to a phenotypic assessment of one or several different drugs when TDM was not available (due to the difficulty of measuring blood levels of certain drugs or because the history of blood tests was missing) or showed unexplained plasma concentrations out of therapeutic range (i.e., to exclude other causes, for example, a lack of compliance, which concerns 10 to 60 % of individuals with mood disorders [35]).

In 34% of cases, the therapeutic failure was attributable to the metabolic profile. These findings are identical to those of Lloret-Linares et al. (34.7%) in a similar psychiatric context [16] and those of Rollason et al. (35.4%) with analgesics [36]. Both of these studies found a trend of a higher implication of metabolic status in ADR than in non-response, whereas we tended to find the opposite. However, our sample size was too small to proceed to statistical analysis.

In 31% of cases, phenotyping excluded the metabolic profile as the cause of the therapeutic problem and was more suggestive of a pharmacodynamic cause. It thus gave the physicians an argument to switch drugs or therapeutic class. In addition, in certain cases, it allowed the physicians to give factual arguments to patients which explained the role of their metabolism in the recurrent drug-related issues encountered.

Thus, phenotyping gave a clear answer to the therapeutic problem in two-thirds of cases. Among these cases, seven of the ten patients for which we had follow-up information showed a clinical improvement. Phenotyping has been of particular assistance in the therapeutic management of two situations: first, when it revealed that low plasma concentration was due to metabolic status rather than poor compliance as suspected by the physicians; second, in a case where phenotyping showed that the recurrence of adverse drug reactions was due to decreased metabolism and not to a systematic psychological apprehension to drugs.

In the remaining third of cases, phenotyping was able to determine the involvement of the metabolic profile in the therapeutic problem with only certain drugs, or was not interpretable. These situations seemed to occur more frequently when both non-response and ADR motivated the phenotyping, indicating multiple successive lines of pharmacotherapy. The multiplication of pharmacotherapy increases the involvement of multiple opposing metabolic pathways and makes it more difficult for phenotyping to give a clear answer, whatever the result. As Lloret-Linares et al. [16] and Rollason et al. [36] did not describe the therapeutic context of non-response or ADR in their cohorts, they might have identified these complex cases in which phenotyping would not be helpful, prior to phenotyping. Thus, we might have to be more careful in the selection of patients before phenotyping.

Only 23% to 56% of the explored CYP/P-gp had normal activity. This value is lower than the normal genotype values found in the literature for Caucasian populations [30,37,38,39]. This reflects phenoconversion due to environmental factors, as reported by previous genotype/phenotype studies [13,16]. In addition, we highlighted that the presence of enzyme inhibitors did not systematically decrease metabolic activity, which is consistent with the literature [40]. This phenomenon was particularly prevalent for “weak” inhibitors. However, these results should be interpreted with caution. First, some molecules might be more potent than others, and our sample size was too small to proceed to a multivariate analysis or a molecule-by-molecule evaluation to identify drugs with higher inhibiting potential. Second, we based our analysis of the qualification of inhibitors and inducers as weak or potent on the tools developed by the Division of Clinical Pharmacology and Toxicology, Geneva University Hospitals [30]. However, these qualifications seem to vary according to the sources, which do not necessarily consider the same metabolic pathways and the same degree of inhibition [4].

Phenotyping is easily implementable in a hospitalization unit. The main limitation is the burden for the patient due to the cocktail intake and the one-to-three blood tests when measuring P-gp. However, based on our patients’ feedback, and as previously described [40], the tolerance for the procedure was generally good. We had to face the objection of only one patient, who was unwilling to take the drug cocktail. Moreover, the phenotyping was generally favorably received by patients as an opportunity to explain the multiple drug failure and to effectively treat their pathology. This had been quantified by a previous study on genotyping, with the majority of patients hopeful that the test would identify the right psychiatric medication for them [41]. Furthermore, for patients referred by community psychiatrists after multiple therapeutic failure, the multidisciplinary involvement and the personalization of treatment open new avenues for recovery. Regarding the burden for the patient, recent findings suggest that endogenous markers might be used instead of drug probes to assess CYP activities [42,43]. If these findings are confirmed, it would open new possibilities for routine phenotyping. In addition, new assay techniques, particularly dried blood spot sampling—which allows the collection of a small volume of blood from the fingertip [5,27]—will ease the burden of the blood tests.

This study has some limitations. First, due to the retrospective approach, we partly or totally lacked some valuable data: the qualitative and quantitative evaluation of adverse drug reactions, drug dosages, medical and medication history, duration since treatment initiation, follow-up data, and patients’ and physicians’ satisfaction feedback. In particular, many data concerning drug-level blood tests were missing, as they were not performed routinely and results were not systematically registered in the medical file. Moreover, patient adherence to treatment was not quantified. Second, we had very small samples which precluded any statistical test. However, this preliminary study was only intended as a descriptive analysis to prepare a future prospective study of our phenotyping.

## 5. Conclusions

CYP and P-gp phenotyping is easily implementable in specialized centers and can provide solutions for complex psychiatric cases. Despite interest in the community, and in contrast to genotyping, only a few studies have been concerned with the clinical outcomes of the phenotypic assessment of metabolic pathways. Our study seems to show a clinical benefit of the phenotypic assessment of the metabolic pathway in clinical practice. However, as this study was only descriptive, these conclusions have to be confirmed by further larger-scale multicentered studies. Notwithstanding small samples and missing data, our study is, to our knowledge, the first to evaluate the consequences of CYP/P-gp phenotyping in terms of therapeutic adaptation and clinical outcomes in psychiatric patients.

## Figures and Tables

**Table 1:** Population and phenotyping characteristics.

Age (year) (mean ± SD)		47.1 ± 14.5
Number of female patients (n, % of the 31 patients)		22 (71%)
Tobacco consumption (n, % of the 31 patients)		9 (29%)
Alcohol consumption >2 glass/day (n, % of the 31 patients)		4 (13%)
Grapefruit consumption (n, % of the 31 patients)		0 (0%)
*Hypericum* consumption (n, % of the 31 patients)		0 (0%)
Type of patient care at the assessment (n, % of the 32 phenotypings)		
	Inpatients	13 (41%)
	Outpatients	19 (59%)
Clinical context (n, % of the 32 phenotypings)		
	Depressive disorder	15 (47%)
	Bipolar disorder	12 (38%)
	Anxiety–depressive syndrome	3 (9%)
	Schizoaffective disorder	1 (3%)
	Neuropathic pain	1 (3%)
Number of drugs implicated (mean ± SD)		7 ± 4.8
Therapeutic problem (n, % of the 32 phenotypings)		
	ADR	10 (31%)
	NR	6 (19%)
	ADR+NR	16 (50%)

ADR: adverse drug reaction; NR: non-response.

**Table 2:** Drugs most implicated in the therapeutic problems that led to a phenotypic assessment.

Drug	Number of Phenotypings	Implication of the Drug in Therapeutic Problems	
		Non-Response	Adverse Drug Reaction
Venlafaxine	17	6	11
Fluoxetine	15	6	9
Paroxetine	10	2	8
Mirtazapine	10	6	4
Clomipramine	10	4	6
Quetiapine	10	1	9
Lithium	10	3	7
Lamotrigine	9	4	5
Aripiprazole	9	3	6
Olanzapine	7	4	3
Sertraline	6	0	6
Escitalopram	6	2	4
Risperidone	6	2	4
Duloxetine	5	1	4
Amitriptyline	5	3	2
Vortioxetine	4	3	1
Chlorpromazine	4	3	1

**Table 3:** Interpretation of the phenotypic assessment, in terms of the involvement of the metabolic profile in the therapeutic problem, depending on the context of the demand and the number of drugs implicated.

		Number of Phenotypings (n)	Involvement of Metabolic Profile in the Therapeutic Problem (n–% of Responses in the Corresponding Therapeutic Problem)					
			Y	N	Y+N	P	NI	P+NI
**Therapeutic problem**	**ADR**	**10**	4 (40%)	4 (40%)	**8 (80%)**	2 (20%)	0 (0%)	**2 (20%)**
	**NR**	**6**	4 (67%)	1 (17%)	**5 (83%)**	0 (0%)	1 (17%)	**1 (17%)**
	**ADR+NR**	**16**	3 (19%)	5 (31%)	**8 (50%)**	5 (31%)	3 (19%)	**8 (50%)**
	**Total**	**32**	11 (34%)	10 (31%)	**21 (66%)**	7 (22%)	4 (13%)	**11 (34%)**
**Number of drugs involved** (mean ± SD)		7.0 ± 4.8	4.5 ± 2.9	8.7 ± 4.5	**6.5 ± 4.2**	8.0 ± 7.1	8.0 ± 3.4	**8.0 ± 5.8**

ADR: adverse drug reaction; N: the phenotyping precluded the metabolic profile as a possible cause of the therapeutic problem; NI: phenotyping results were not interpretable; NR: non-response; P: the metabolic profile might have been partially responsible for the therapeutic problem; Y: the metabolic profile is compatible with under- or overdosing that might have caused the therapeutic problem.

**Table 4:** Cytochrome (CYP) and P-glycoprotein (P-gp) measured activity in the presence and absence of an inhibitor.

			Number of Phenotypic Assessments (n)	CYP/P-gp Measured Activity (% of the Phenotypic Assessments)			
				Decreased/PM	Normal/IM	Increased/EM	UM
**CYP1A2**			**30**	**23%**	**43%**	**33%**	
	In the presence of an inhibitor		8	38%	50%	13%	
		*Weak*	*8*	*38%*	*50%*	*13%*	
		*Potent*					
**CYP2B6**			**30**	**0%**	**40%**	**60%**	
	In the presence of an inhibitor		1		100%		
		*Weak*	*1*		*100%*		
		*Potent*					
**CYP2C9**			**30**	**33%**	**23%**	**43%**	
	In the presence of an inhibitor		8		25%	75%	
		*Weak*	*4*		*25%*		
		*Potent*	*4*		*25%*		
**CYP2C19**			**32**	**38%**	**53%**	**9%**	
	In the presence of an inhibitor		9	89%	11%		
		*Weak*	*6*	*83%*	*17%*		
		*Potent*	*3*	*100%*			
**CYP2D6**			**32**	**22%**	**56%**	**16%**	**6%**
	In the presence of an inhibitor		29	21%	62%	17%	
		*Weak*	*17*	*6%*	*65%*	*29%*	
		*Potent*	*12*	*42%*	*58%*		
**CYP3A4**			**30**	**33%**	**43%**	**23%**	
	In the presence of an inhibitor		8	13%	75%	13%	
		*Weak*	*8*	*13%*	*75%*	*13%*	
		*Potent*					
**P-gp**			**30**	**57%**	**40%**	**3%**	
	In the presence of an inhibitor		11	73%	27%		
		*Weak*	*4*	*75%*	*25%*		
		*Potent*	*7*	*71%*	*29%*		

EM: extensive metabolizer; IM: intermediate metabolizer; PM: poor metabolizer; UM: ultrarapid metabolizer.

## Data Availability

The data that support the findings of this study are available from the corresponding author upon reasonable request.

## Supplementary Materials

The following are available online at https://www.mdpi.com/article/10.3390/jpm12111869/s1. Table S1: Drug cocktail details. Table S2: Patient individual characteristics and key assessments. Table S3: Drugs implicated in the therapeutic problem that led to the 32 phenotypings and the CYP/P-gp for which they are a substrate. Table S4: Effect of CYP/P-gp inhibitor intake on the activity of the CYP/P-gp they inhibit. Table S5: Effect of CYP/P-gp inducer intake on the activity of the CYP/P-gp they induce.
