# Supplementary material for: Cytochromes P450 and P-Glycoprotein Phenotypic Assessment to Optimize Psychotropic Pharmacotherapy: A Retrospective Analysis of Four Years of Practice in Psychiatry
Source: J Pers Med. 2022 Nov 8;12(11):1869. doi: 10.3390/jpm12111869 (PMC9693601; doi:10.3390/jpm12111869)
Supplement: Supplementary file 1 [file jpm-12-01869-s001.zip › jpm-1965093-supplementary.pdf]

SUPPLEMENTAL DATA

Table S1. Drug cocktail details

**Probe drug**	**Metabolite**	**CYP/P-gp**	**Dosage (mg)**	**Form**	**Route of administration**
**Caffeine**	paraxanthine	CYP1A2	50	oral solution	oral
**Bupropion**	4-hydroxybupropion	CYP2B6	150	tablet	oral
**Flurbiprofen**	4-hydroxyflurbiprofen	CYP2C9	50	tablet	oral
**Omeprazole**	5-hydroxyomeprazole	CYP2C19	10	capsule	oral
**Dextromethorphan**	dextrorphan	CYP2D6	10	oral solution	oral
**Midazolam**	1-hydroxymidazolam	CYP3A4	1	injectable solution	oral
**Fexofenadine**		P-gp	120	capsule	oral

Table S2: Patient individual characteristics and key assessments.

$:Phenotyping 1 and 2 concerned the same patient; ↘: decreased/Poor Metabolizer; ↗: increased/Extensive Metabolizer; ↗↗: Ultrarapid Metabolizer; ADR: Adverse Drug Reaction; N: the phenotyping precluded the metabolic profile as a possible cause of the therapeutic problem; NA: not assessed; NI: phenotyping results were not interpretable; Norm.: Normal/intermediate metabolizer; NR: Non-Response; P: the metabolic profile might have been partially responsible for the therapeutic problem; Y: the metabolic profile is compatible with an under- or overdosing that might have caused the therapeutic problem

Phenotyping	Age (year)	Gender	Tobacco consumption	Alcohol consumption (>2 glass/day)	Clinical context	Therapeutic problem	Number of drugs involved	Assessed activities							Involvement of metabolic profile in the therapeutic problem	Follow-up	Consequences
								1A2	2B6	2C9	2C19	2D6	3A4	P-gp			
1$	31	M	No	No	Depressive disorder	ADR	5	↘	↗	↘	↘	↘	Norm.	↘	Y		
2$					Neuropathic pain	NR	1	↗	↗	↘	Norm.	↘	Norm.	NA	Y		
3	53	F	No	No	Depressive disorder	ADR	6	↘	↗	↘	Norm.	↗	↘	NA	P		
4	69	M	No	No	Depressive disorder	ADR	1	Norm.	↗	↗	↘	Norm.	↘	↘	Y		
5	39	F	No	No	Bipolar disorder	ADR	7	Norm.	↗	↗	↘	↗	↗	↘	N		
6	49	F	No	No	Bipolar disorder	ADR+NR	12	↘	↗	↘	Norm.	Norm.	↘	↘	NI		
7	25	M	Yes	No	Schizoaffective disorder	NR	1	↗	NA	NA	Norm.	↗↗	↗	↗	Y	Increased clozapine dosage	Clinical improvement
8	28	F	Yes	Yes	Depressive disorder	ADR+NR	2	NA	↗	Norm.	↘	Norm.	Norm.	Norm.	N	Pharmacological class switch	No improvement
9	32	F	Yes	No	Bipolar disorder	ADR+NR	3	NA	↗	↗	↗	↗↗	↗	Norm.	P		
10	57	F	No	No	Depressive disorder	ADR	9	↘	Norm.	Norm.	↘	Norm.	Norm.	Norm.	Y	Drug switch	Clinical improvement
11	60	F	Yes	No	Depressive disorder	ADR+NR	7	↗	↗	↗	↗	Norm.	↘	↘	NI		

12	35	F	No	No	Depressive disorder	ADR+NR	3	Norm.	Norm.	↘	↘	↘	Norm.	↘	P		
13	47	F	No	No	Bipolar disorder	ADR+NR	5	Norm.	NA	NA	Norm.	↘	↗	Norm.	Y	Increased clozapine dosage	No improvement
14	42	F	No	No	Bipolar disorder	NR	4	Norm.	↗	↘	Norm.	Norm.	Norm.	↘	Y		
15	75	F	No	No	Depressive disorder	ADR+NR	4	Norm.	↗	↘	Norm.	Norm.	↗	↘	NI		
16	49	F	No	Yes	Bipolar disorder	ADR+NR	7	Norm.	Norm.	↗	↘	↘	Norm.	↘	P		
17	65	F	No	No	Depressive disorder	NR	4	Norm.	↗	↗	Norm.	Norm.	Norm.	↘	Y		
18	59	M	No	No	Bipolar disorder	ADR+NR	8	Norm.	↗	Norm.	Norm.	↗	↘	↘	Y	Drug switch	Clinical improvement
19	41	F	No	No	Depressive disorder	ADR	3	↘	Norm.	↗	↘	Norm.	↘	Norm.	Y	Drug switch	Clinical improvement
20	42	F	No	No	Bipolar disorder	ADR+NR	23	Norm.	Norm.	Norm.	Norm.	↗	NA	↘	P		
21	35	M	No	Yes	Depressive disorder	ADR+NR	17	Norm.	Norm.	Norm.	Norm.	Norm.	Norm.	↘	N		
22	61	F	No	No	Depressive disorder	NR	15	↗	Norm.	↗	Norm.	Norm.	Norm.	↘	N	Pharmacological class switch	Clinical improvement
23	51	F	No	No	Depressive disorder	ADR+NR	10	↗	↗	Norm.	↘	↗	NA	↘	N	Pharmacological class switch	Clinical improvement
24	65	F	No	No	Bipolar disorder	ADR+NR	8	↘	Norm.	↗	↘	↘	Norm.	↘	Y	Drug switch	Clinical improvement
25	50	F	Yes	No	Bipolar disorder	NR	9	↘	↗	↗	Norm.	Norm.	↗	Norm.	NI		
26	32	F	No	No	Depressive disorder	ADR	9	Norm.	Norm.	↗	↘	Norm.	Norm.	Norm.	N		
27	25	M	Yes	No	Bipolar disorder	ADR+NR	4	↗	Norm.	↗	Norm.	↘	Norm.	Norm.	P		
28	58	F	Yes	No	Anxiety-depressive syndrome	ADR+NR	5	↗	Norm.	↗	Norm.	Norm.	↘	Norm.	N		

29	49	F	Yes	No	Bipolar disorder	ADR	8	↗	↗	Norm.	Norm.	Norm.	↗	Norm.	N		
30	37	M	No	No	Anxiety-depressive syndrome	ADR	10	Norm.	Norm.	↘	↘	Norm.	↘	Norm.	P		
31	27	M	No	No	Depressive disorder	ADR+NR	8	↗	↗	↘	↗	Norm.	↘	↘	N	No change	No improvement
32	71	M	Yes	Yes	Anxiety-depressive syndrome	ADR	6	↗	↗	↘	Norm.	Norm.	↘	Norm.	N		

Table S3. Drugs implicated in the therapeutic problem that led to the 32 phenotyping and the CYP/P-gp they are substrate.

.Phenotyping	Implicated drug	CYP1A2 Substrate	CYP2B6 Substrate	CYP2C9 Substrate	CYP2C19 Substrate	CYP2D6 Substrate	CYP3A4 Substrate	P-gp Substrate
1	Venlafaxine					**Major**	Minor	**Major**
	Mirtazapine	Minor				**Major**	**Major**	
	Olanzapine	**Major**				Minor		Minor
	Paroxetine					**Major**		
	Quetiapine					Minor	**Major**	
2	Ketamine		**Major**					
3	Fluoxetine			**Major**	Minor	**Major**	Minor	
	Quetiapine					Minor	**Major**	
	Lithium							
	Clomipramine	**Major**			Minor	**Major**	**Major**	
	Tramadol		**Major**			Minor	Major	
	Codeine					Minor	**Major**	
4	Fluoxetine			**Major**	Minor	**Major**	Minor	
5	Lithium							
	Chlorpromazine							
	Lorazepam							
	Prazepam							
	Fluoxetine			**Major**	Minor	**Major**	Minor	
	Paroxetine					**Major**		
	Fluvoxamine	Minor				**Major**		**Major**
6	Fluoxetine			**Major**	Minor	**Major**	Minor	
	Lamotrigine							
	Lithium							
	Tianeptine							

	Clomipramine	**Major**			Minor	**Major**	**Major**	
	Risperidone					**Major**	Minor	**Major**
	Venlafaxine					**Major**	Minor	**Major**
	Mirtazapine	Minor				**Major**	**Major**	
	Paroxetine					**Major**		
	Mianserin	**Major**				**Major**	Minor	
	Tramadol		**Major**			Minor	Major	
	Opium							
7	Clozapine	**Major**		Minor	**Major**	Minor	**Major**	**Major**
8	Venlafaxine					**Major**	Minor	**Major**
	Fluoxetine			**Major**	Minor	**Major**	Minor	
9	Valproate			Minor				
	Olanzapine	**Major**				Minor		Minor
	Lithium							
10	Clomipramine	**Major**			Minor	**Major**	**Major**	
	Sertraline		**Major**	Minor	Minor	Minor	Minor	Minor
	Venlafaxine					**Major**	Minor	**Major**
	Fluoxetine			**Major**	Minor	**Major**	Minor	
	Aripiprazole					**Major**	**Major**	**Major**
	Duloxetine	**Major**				**Major**		
	Lorazepam							
	Lamotrigine							
	Paroxetine					**Major**		
11	Agomelatine	**Major**		Minor	Minor			
	Duloxetine	**Major**				**Major**		
	Loxapine							
	Lorazepam							
	Paroxetine					**Major**		
	Clomipramine	**Major**			Minor	**Major**	**Major**	
	Fluoxetine			**Major**	Minor	**Major**	Minor	
12	Lithium							
	Paroxetine					**Major**		
	Prazepam							
13	Diazepam				**Major**		**Major**	
	Oxcarbazepine	Minor		Minor			**Major**	
	Lithium							
	Aripiprazole					**Major**	**Major**	**Major**
	Chlorpromazine							
14	Lamotrigine							
	Lithium							
	Mirtazapine	Minor				**Major**	**Major**	

	Venlafaxine					**Major**	Minor	**Major**
15	Diazepam				**Major**		**Major**	
	Clomipramine	**Major**			Minor	**Major**	**Major**	
	Primidone			**Major**	**Major**			
	Venlafaxine					**Major**	Minor	**Major**
16	Alimemazine							
	Amisulpride							
	Aripiprazole					**Major**	**Major**	**Major**
	Clozapine	**Major**		Minor	**Major**	Minor	**Major**	**Major**
	Quetiapine					Minor	**Major**	
	Risperidone					**Major**	Minor	**Major**
	Venlafaxine					**Major**	Minor	**Major**
17	Quetiapine					Minor	**Major**	
	Venlafaxine					**Major**	Minor	**Major**
	Mirtazapine	Minor				**Major**	**Major**	
	Olanzapine	**Major**				Minor		Minor
18	Mirtazapine	Minor				**Major**	**Major**	
	Aripiprazole					**Major**	**Major**	**Major**
	Vortioxetine							
	Lormetazepam							
	Cyamemazine							
	Pramipexole							
	Fluoxetine			**Major**	Minor	**Major**	Minor	
	Venlafaxine					**Major**	Minor	**Major**
19	Duloxetine	**Major**				**Major**		
	Escitalopram							
	Agomelatine	**Major**		Minor	Minor			
20	Valproate			Minor				
	Lithium							
	Quetiapine					Minor	**Major**	
	Aripiprazole					**Major**	**Major**	**Major**
	Lamotrigine							
	Carbamazepine	Minor		Minor			**Major**	
	Risperidone					**Major**	Minor	**Major**
	Olanzapine	**Major**				Minor		Minor
	Topiramate							
	Fluoxetine			**Major**	Minor	**Major**	Minor	
	Sertraline		**Major**	Minor	Minor	Minor	Minor	Minor
	Escitalopram							
	Citalopram				**Major**	Minor	**Major**	**Major**
	Venlafaxine					**Major**	Minor	**Major**

	Mirtazapine	Minor				**Major**	**Major**	
	Pramipexole							
	Liothyronine							
	Paroxetine					**Major**		
	Fluvoxamine	Minor				**Major**		**Major**
	Clomipramine	**Major**			Minor	**Major**	**Major**	
	Amitriptyline	Minor		Minor		**Major**	Minor	**Major**
	Trimipramine			Minor	Minor	**Major**		**Major**
	Vortioxetine							
21	Escitalopram							
	Sertraline		**Major**	Minor	Minor	Minor	Minor	Minor
	Fluoxetine			**Major**	Minor	**Major**	Minor	
	Venlafaxine					**Major**	Minor	**Major**
	Amitriptyline	Minor		Minor		**Major**	Minor	**Major**
	Mirtazapine	Minor				**Major**	**Major**	
	Carbamazepine	Minor		Minor			**Major**	
	Lamotrigine							
	Lithium							
	Bupropion		**Major**					
	Cyamemazine							
	Loxapine							
	Quetiapine					Minor	**Major**	
	Eslicarbazepine							
	Clobazam				**Major**		**Major**	
	Methylphenidate							
	Dexamphetamine							
22	Phenelzine							
	Aripiprazole					**Major**	**Major**	**Major**
	Fluoxetine			**Major**	Minor	**Major**	Minor	
	Amitriptyline	Minor		Minor		**Major**	Minor	**Major**
	Venlafaxine					**Major**	Minor	**Major**
	Dosulepine							
	Vortioxetine							
	Duloxetine	**Major**				**Major**		
	Clomipramine	**Major**			Minor	**Major**	**Major**	
	Levomepromazine					**Major**		
	Haloperidol					**Major**	**Major**	
	Risperidone					**Major**	Minor	**Major**
	Pregabalin							
	Valpromide			Minor				
	Carbamazepine	Minor		Minor			**Major**	

23	Milnacipran							
	Venlafaxine					**Major**	Minor	**Major**
	Sertraline		**Major**	Minor	Minor	Minor	Minor	Minor
	Escitalopram							
	Agomelatine	**Major**		Minor	Minor			
	Duloxetine	**Major**				**Major**		
	Vortioxetine							
	Paroxetine					**Major**		
	Lamotrigine							
	Bromazepam							
24	Venlafaxine					**Major**	Minor	**Major**
	Escitalopram							
	Mirtazapine	Minor				**Major**	**Major**	
	Pramipexole							
	Lithium							
	Clomipramine	**Major**			Minor	**Major**	**Major**	
	Olanzapine	**Major**				Minor		Minor
	Fluoxetine			**Major**	Minor	**Major**	Minor	
25	Clozapine	**Major**		Minor	**Major**	Minor	**Major**	**Major**
	Lithium							
	Valproate			Minor				
	Olanzapine	**Major**				Minor		Minor
	Aripiprazole					**Major**	**Major**	**Major**
	Quetiapine					Minor	**Major**	
	Diazepam				**Major**		**Major**	
	Risperidone					**Major**	Minor	**Major**
	Chlorpromazine							
26	Paroxetine					**Major**		
	Venlafaxine					**Major**	Minor	**Major**
	Mianserin	**Major**				**Major**	Minor	
	Fluoxetine			**Major**	Minor	**Major**	Minor	
	Mirtazapine	Minor				**Major**	**Major**	
	Quetiapine					Minor	**Major**	
	Loxapine							
	Aripiprazole					**Major**	**Major**	**Major**
	Alprazolam						**Major**	
27	Quetiapine					Minor	**Major**	
	Chlorpromazine							
	Amisulpride							
	Clozapine	**Major**		Minor	**Major**	Minor	**Major**	**Major**
28	Escitalopram							
	Fluoxétine			**Major**	Minor	**Major**	Minor	

	Sertraline		**Major**	Minor	Minor	Minor	Minor	Minor
	Alprazolam						**Major**	
	Mélatonine							
29	Risperidone					**Major**	Minor	**Major**
	Oxcarbazepine	Minor		Minor			**Major**	
	Topiramate							
	Quetiapine					Minor	**Major**	
	Carbamazepine	Minor		Minor			**Major**	
	Aripiprazole					**Major**	**Major**	**Major**
	Amisulpride							
	Lamotrigine							
30	Sertraline		**Major**	Minor	Minor	Minor	Minor	Minor
	Fluoxetine			**Major**	Minor	**Major**	Minor	
	Venlafaxine					**Major**	Minor	**Major**
	Amitriptyline	Minor		Minor		**Major**	Minor	**Major**
	Citalopram				**Major**	Minor	**Major**	**Major**
	Clomipramine	**Major**			Minor	**Major**	**Major**	
	Paroxetine					**Major**		
	Amisulpride							
	Valproate			Minor				
	Lamotrigine							
31	Lamotrigine							
	Venlafaxine					**Major**	Minor	**Major**
	Olanzapine	**Major**				Minor		Minor
	Ropinirole							
	Alprazolam						**Major**	
	Zolpidem	Minor		Minor	Minor	Minor	**Major**	
	Zopiclone						**Major**	
	Bromazepam							
32	Amitriptyline	Minor		Minor		**Major**	Minor	**Major**
	Mianserin	**Major**				**Major**	Minor	
	Clomipramine	**Major**			Minor	**Major**	**Major**	
	Mirtazapine	Minor				**Major**	**Major**	
	Pramipexole							
	Oxazepam							
Phenotyping	Implicated drug	CYP1A2 Substrate	CYP2B6 Substrate	CYP2C9 Substrate	CYP2C19 Substrate	CYP2D6 Substrate	CYP3A4 Substrate	P-gp Substrate
Total drugs	224							
Total substrates		56	9	47	52	128	116	59
Major substrates		*32*	*9*	*16*	*16*	*95*	*64*	*46*
Minor substrates		*24*	*0*	*31*	*36*	*33*	*52*	*13*

Table S4. Effect of CYP/P-gp inhibitors intake on the activity of the CYP/P-gp they inhibit.

The number in the "measured activity" column correspond to the number of phenotyping in which the CYP/P-gp activity that the drug was supposed to inhibit was measured decreased (↓), normal (N) or increased (↑). Underscored numbers indicate the inhibitor is potent for this CYP/P-gp.

**CYP/P-gp inhibitor**	**Number of phenotyping with the inhibitor** (n)	**CYP1A2**			**CYP2B6**			**CYP2C19**			**CYP2C9**			**CYP2D6**			**CYP3A4**			**P-gp**			**Total**		
		**Measured activity**			**Measured activity**			**Measured activity**			**Measured activity**			**Measured activity**			**Measured activity**			**Measured activity**			**Measured activity**		
		↓	N	↑	↓	N	↑	↓	N	↑	↓	N	↑	↓	N	↑	↓	N	↑	↓	N	↑	↓	N	↑
Alimemazine	1														1					1			1	1	
Chlorpromazine	2													1	1								1	1	
Clomipramine	2														2									2	
Desogestrel	1							1										1			1		1	2	

Doxycycline	1																		1						1
Doxylamine	1														1									1	
Escitalopram	1														1									1	
Ethinylestradiol	1																	1						1	
Fluoxetine	4	1	2					4				1	3	2	2		1	3		3	1		11	9	3
Hydroxyzine	3														2	1								2	1
Lansoprazole	2							2												2			4		
Levomepromazine	1													1									1		
Loxapine	1		1													1				1			1	1	1
Metoclopramide	1															1									1
Naltrexone	1																			1			1		
Nevirapine	1	1													1								1	1	
Oxcarbazepine	1								1															1	
Paroxetine	2		2											1	1					1	1		2	4	
Phenelzine	1			1											1			1						2	1
Phloroglucinol	1																			1			1		
Risperidone	1															1									1
Sertraline	1	1				1									1								1	2	
Valproate	4											1	3											1	3
Venlafaxine	6													1	4	1							1	4	1
Vortioxetine	1																			1			1		
**Total**	**42**	**3**	**5**	**1**		**1**		**7**	**1**			**2**	**6**	**6**	**18**	**5**	**1**	**6**	**1**	**11**	**3**		**28**	**36**	**13**

Table S5. Effect of CYP/P-gp inducers intake on the activity of the CYP/P-gp they induce.

The number in the "measured activity" column correspond to the number of phenotyping in which the CYP/P-gp activity that the drug was supposed to induce was measured decreased (↓), normal (N) or increased (↑). Underscored numbers indicate the inducer is potent for this CYP/P-gp.

**CYP/P-gp inducer**	**Number of phenotyping with the inducer** (n)	**CYP1A2**			**CYP2B6**			**CYP2C19**			**CYP2C9**			**CYP2D6**			**CYP3A4**			**P-gp**			**Total**		
		**Measured activity**			**Measured activity**			**Measured activity**			**Measured activity**			**Measured activity**			**Measured activity**			**Measured activity**			**Measured activity**		
		↑	N	↓	↑	N	↓	↑	N	↓	↑	N	↓	↑	N	↓	↑	N	↓	↑	N	↓	↑	N	↓
Lansoprazole	2			2																					2
Nevirapine	1						1												1						2
Oxcarbazepine	1																1						1		
Primidone	2				2				1		1						2				1	1	5	2	1
**Total**	**6**			**2**	**2**		**1**		**1**		**1**						**3**		**1**		**1**	**1**	**6**		**5**
